# Maternal sleep deprivation during pregnancy induced offspring germ cells loss through ferroptosis

**DOI:** 10.1038/s41420-025-02839-5

**Published:** 2025-11-24

**Authors:** Qingchun Liu, Jiamao Yan, Han Wang, Tao Wang, Kai Zhang, Aiying Li, Junjie Wang, Teng Zhang, Wei Shen, Lan Li

**Affiliations:** 1 College of Animal Science and Technology, Shandong Engineering Research Center for Protection of Livestock and Poultry Genetic Resources and Biological Breeding, Qingdao Agricultural University, 266109, Qingdao, China; 2 State Key Laboratory of Reproductive Regulation and Breeding of Grassland Livestock (R2BGL), College of Life Sciences, Inner Mongolia University, 010070, Hohhot, China; 3 College of Life Sciences, Qingdao Agricultural University, 266109, Qingdao, China

**Keywords:** Infertility, Apoptosis, Transcriptomics

## Abstract

Primordial follicles form the non-renewable germline reserve in female mammals; their limited number is a key determinant of reproductive lifespan and is crucial for female fertility. Insufficient or parasomnia sleep is common in females, yet little attention is paid to the impact of such sleep disorders during pregnancy on the health of offspring, particularly with regard to ovarian development. In this study, a sleep deprivation (SD) model was established with pregnant mice from 6.5 to 13.5 days of pregnancy. The pregnant mice were subjected to 18 h of SD per day, and the effects on the reproductive function of female offspring and the potential mechanisms were explored. Results showed that SD not only affected maternal weight gain and hormone levels, but also significantly decreased the number of germ cells in female offspring. Furthermore, maternal SD may impact the development of female germ cells by interfering with meiotic progression and inhibiting primordial follicle formation. Importantly, in vitro and in vivo experiments demonstrated that inhibition of ferroptosis improved germ cell loss in offspring caused by maternal SD. Thus, for the first time, our study verified a potential link between germ cell loss during primordial follicle formation and ferroptosis; in theory, this may provide treatment options for reproductive damage in offspring caused by maternal sleep disorders during pregnancy.

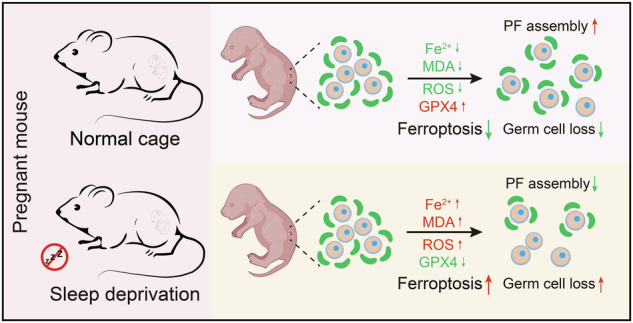

## Introduction

Sleep is crucial for human physical and mental health. Numerous studies confirm that insufficient sleep can negatively affect health, including cognitive function decline [1], emotional disorders [2], increased risk of cardiovascular and cerebrovascular diseases [3, 4], and impaired immune function [5]. Recent surveys indicate that up to 43.9% of women suffer from insomnia during pregnancy [2], and nearly two-thirds of pregnant women experience reduced sleep duration and decreased sleep quality [6, 7]. Furthermore, studies indicate that women with insufficient sleep during pregnancy may face a longer labor, more intense pain and discomfort, as well as higher risks of preterm birth and cesarean section [8]. In addition, lack of sleep during pregnancy may impact fetal development and postpartum health [9]. Although many studies show that maternal sleep deprivation (SD) during pregnancy affects offspring health, the effects of SD during pregnancy on offspring ovarian development are largely unknown.

Ovarian follicles are fundamental, non-renewable reproductive units in female mammals, and the primordial follicle (PF) is the starting point of follicle development [10]. In mice, primordial germ cells (PGCs) migrate to the developing gonads at embryonic stage 10.5 (E10.5) and undergo mitosis to produce germ cell cysts (or nests) [11]. Subsequently, these female germ cells begin to initiate meiosis at E13.5, becoming gradually differentiated into oocytes. At E17.5, pre-granulosa cells begin to invade the cyst, a process which is accompanied by a massive loss of germ cells, and PFs––oocytes surrounded by a single layer of granulosa cells––are formed [12, 13]. In most mammals, the primordial follicle pool is established perinatally, and intricate mechanisms ensure that the majority of PFs remain quiescent and maintain this state for long periods, even decades [14]. Currently, it is generally believed that the PF reserve is a non-renewable resource that gradually declines after puberty until exhaustion (in humans) at menopause [15], and that the size of the PF reserve determines the length of reproductive life [16]. Pregnancy is a critical time for offspring PF development, during which the mother is highly sensitive to environmental factors, and adverse environmental stimuli may have long-term and irreversible effects on offspring reproductive development [17, 18]. Thus, good quality sleep during pregnancy is important for maintaining the reproductive health of offspring.

Ferroptosis, a complex process regulated by multiple factors, is an oxidized and iron-dependent form of regulated cell death; it is generally characterized by increased intracellular free iron content, the accumulation of reactive oxygen species (ROS), and lipid peroxidation [19, 20]. Cells undergoing ferroptosis often exhibit mitochondrial dysfunction; this is characterized by a decrease in mitochondrial volume and membrane density, a reduction or disappearance of cristae structures, rupture of the outer membrane, and other morphological changes [21]. Iron ions play a central role, with increased levels of intracellular ferrous iron (Fe²⁺) driving the production of toxic ROS, thereby inducing ferroptosis [22]. A diminished cellular response to iron ions may facilitate iron accumulation and lipid peroxidation, consequently increasing the risk of ferroptosis [23]. In addition to imbalanced iron homeostasis and amino acid antioxidant system, the accumulation of lipid peroxides can disrupt cell membrane integrity, also ultimately leading to cellular ferroptosis [24, 25]. As an antioxidant enzyme, glutathione peroxidase 4 (GPX4) is a known key factor in regulating ferroptosis; GPX4 inactivation can lead to peroxide accumulation within cells, which triggers the process [26].

Several recent studies report the impact of SD on ovarian development [27–29]; however, the effects of maternal SD during pregnancy on early oogenesis in offspring are largely unknown. Germ cell development is a continuous process, and the effects of maternal SD on fetal development are often cumulative and delayed. Currently, there is no consensus regarding the timing of SD during pregnancy, as studies have examined various periods, including early, mid, or late gestation, as well as the entire gestational period [30–34]. The E6.5–E13.5 period covers critical stages of germ cell development, including migration and meiotic initiation, thus avoiding the potential embryonic instability associated with earlier stages (before E6.5) and the increased risk of miscarriage caused by SD devices in late gestation. Preliminary experiments indicate that this time frame strikes a balance between maternal tolerance and offspring phenotype, ensuring the reproducibility and reliability of the experiments. Moreover, early defects in fetal germ-cell development—such as those induced by maternal harmful exposure or meiotic DNA-damage responses—are known to perturb primordial follicle formation, aligning with our objective [35, 36]. Therefore, we established a maternal SD model from E6.5 to E13.5 in mice and analyzed its effects on primordial follicle assembly in offspring. Our findings offer a new perspective on the profound potential impact of sleep quality during pregnancy on the reproductive health of offspring.

## Results

### Maternal sleep deprivation during pregnancy impaired ovarian development in offspring

**Fig. 1:**
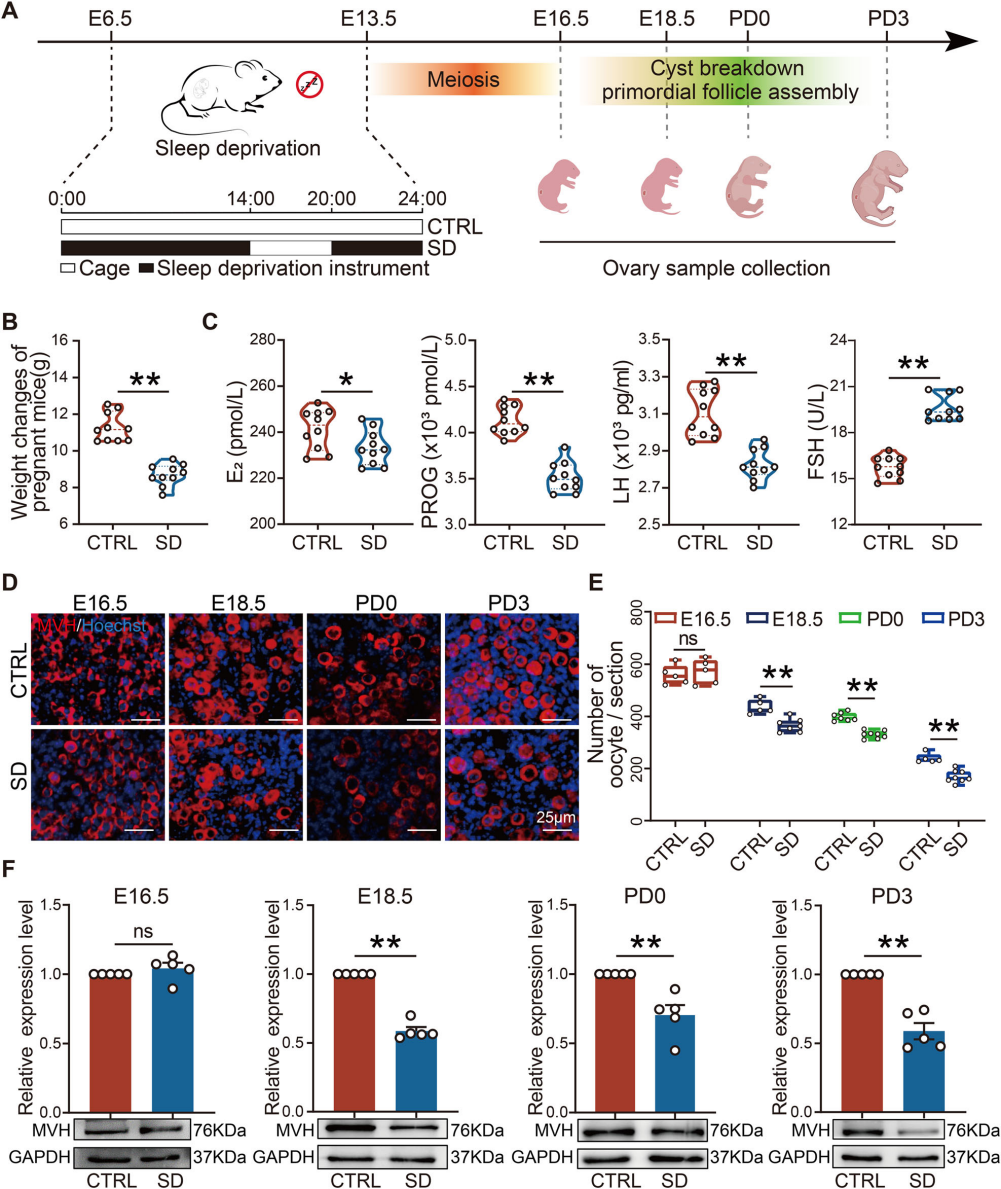
Maternal sleep deprivation during pregnancy impaired ovarian development in offspring. **A** Schematic diagram of the maternal sleep deprivation (SD) during pregnancy experiment. **B** Weight changes in CTRL and SD pregnant mice during SD (*n* = 10). **C** Blood serum concentrations of E2, PROG, LH, and FSH in pregnant mice at the end of the experimental time (*n* = 10). **D** Representative IF for MVH (red) in ovaries of the four experimental groups, nuclei stained with Hoechst 33342 (blue). Scale bar = 25 µm. **E** Number of MVH-positive oocytes in the four experimental groups (*n* ≥ 5). **F** WB results showed the representative images and relative expression levels of MVH protein in E16.5, E18.5 genital ridge, and PD0, PD3 ovaries (*n* = 5).

To explore the potential impact of sleep deprivation (SD) during pregnancy on ovarian development in female offspring, pregnant mice (day 6.5 to 13.5 of gestation) were subjected to 18 h of SD per day (Fig. 1A). During this process, we recorded the body weight change of pregnant mice and found that the weight gain of SD mice was significantly less than that of CTRL (Fig. 1B). Serum hormone analysis revealed that the levels of maternal E2, PROG, and LH in SD were lower than those in CTRL, while FSH was higher (Fig. 1C). To further analyze the impact of SD during pregnancy on the development of offspring’s ovaries, we collected ovarian samples from mice before birth (E16.5, E18.5) and after birth (PD0, PD3), and performed immunohistofluorescence staining (Fig. 1D). Mouse Vasa homolog (MVH, also known as DDX4) is specifically expressed in germ cells and is an important indicator for assessing their quantity and condition [37, 38]. MVH expression was checked and results showed comparable germ cell numbers in CTRL and SD at E16.5. However, the numbers of germ cell in SD at E18.5, PD0, and PD3 were significantly less than those in CTRL (Fig. 1E). There was no significant difference in the protein expression of MVH between CTRL and SD at E16.5, but they were significantly lower in SD at E18.5, PD0, and PD3 than in CTRL (Fig. 1F). These results indicated that maternal SD during pregnancy led to the loss of germ cells in female offspring.

### Maternal sleep deprivation during pregnancy disrupted germ cell meiosis progression in female fetal mice

**Fig. 2:**
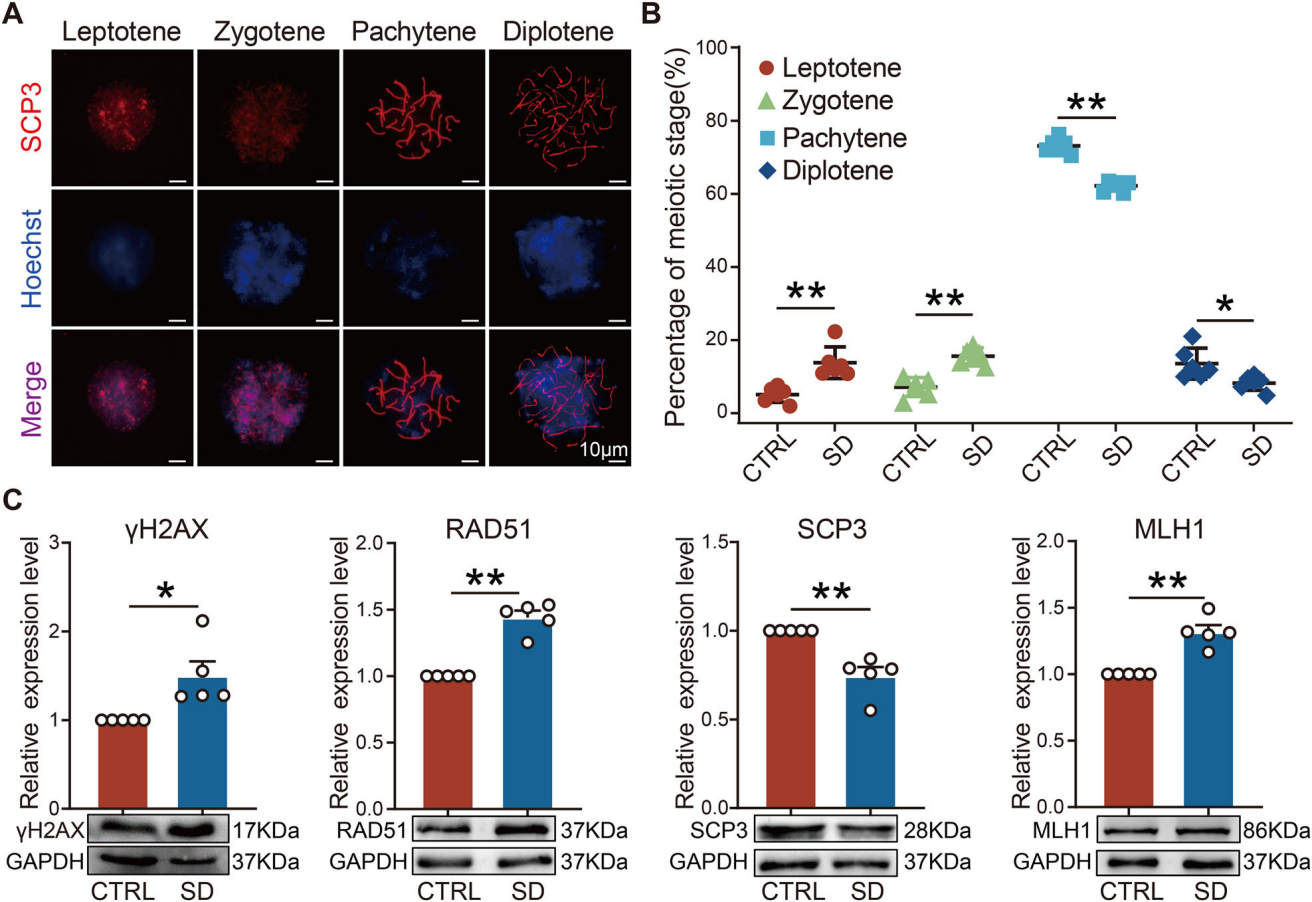
Maternal sleep deprivation during pregnancy affected fetal meiosis. **A** Representative immunofluorescence image of E16.5 oocyte SCP3 (red) and nucleus (blue) staining. Scale bar = 10 μm. **B** The percentage of oocytes at different stages of prophase I of meiosis (*n* = 6). **C** Representative WB images and relative expression levels of γ-H2AX, RAD51, SCP3, and MLH1 proteins in E16.5 genital ridges (*n* = 5).

Since germ cell loss was observed mainly in female offspring after E16.5, we further explored the specific impact of maternal SD on meiosis in female offspring using genital ridge samples at E16.5 (Fig. 2A). The results showed that, compared to CTRL, the SD group had a higher proportion of cells at leptotene and zygotene stages, and a lower proportion at pachytene and diplotene stages (Fig. 2B). During meiosis, DNA double-strand breaks (DSBs) are a key event in initiating homologous chromosome pairing and recombination; DSBs repair is crucial for ensuring the accurate transmission of genetic material. γH2AX is an important marker of the DNA damage response, while RAD51 is a key protein in homologous recombination repair, and MLH1 is a key protein in mismatch repair. We detected the expression levels of these key proteins to assess the repair of DNA damage during meiosis. Western blot results further revealed a downward trend in the expression of Synaptonemal complex protein 3 (SCP3) in SD, while the expression levels of γH2AX, RAD51, and MLH1, were elevated (Fig. 2C). These findings suggest that SD in pregnant mice may disrupted normal meiotic progression in female offspring, thus affecting germ cell development.

### Maternal sleep deprivation during pregnancy inhibited primordial follicle formation in offspring

**Fig. 3:**
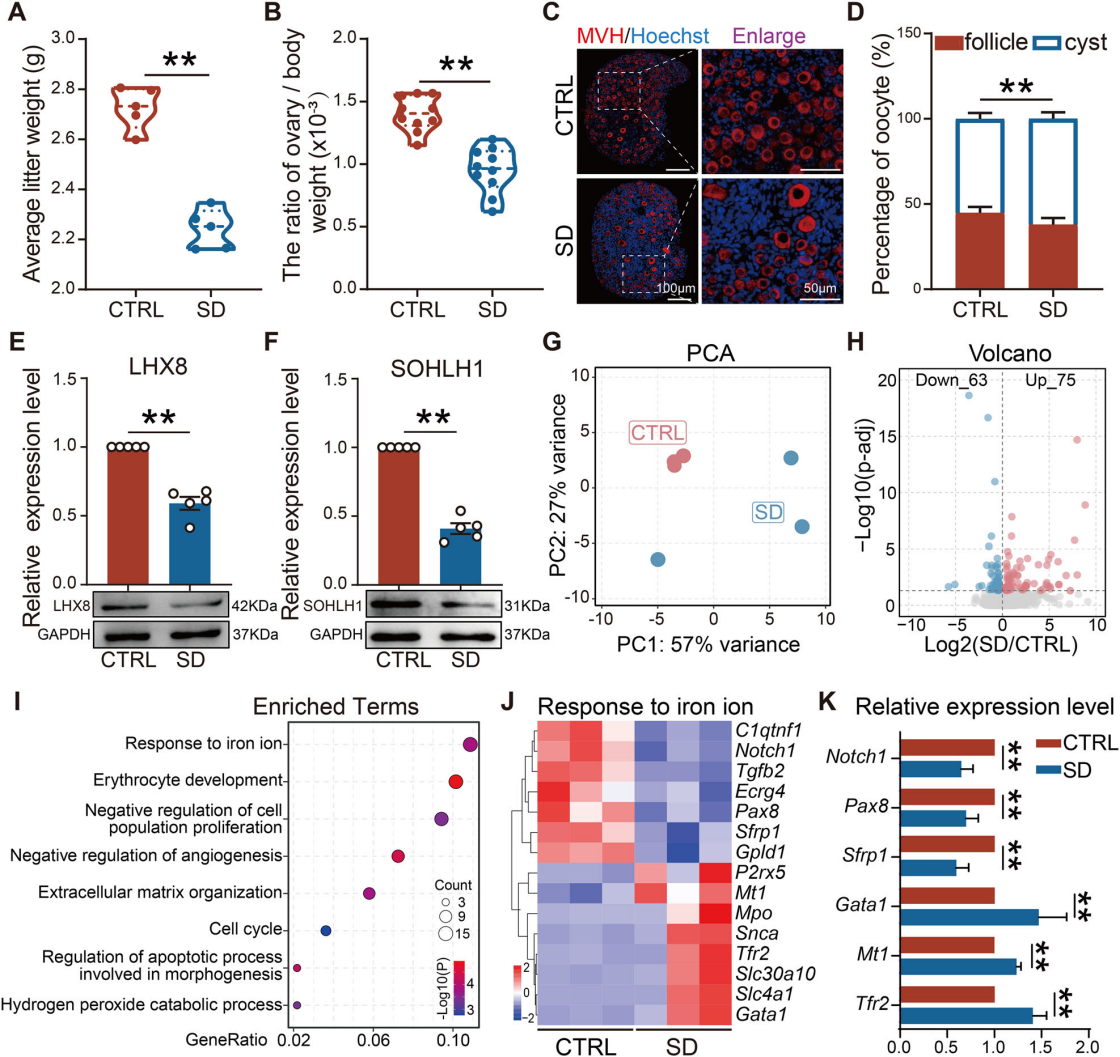
Maternal sleep deprivation during pregnancy inhibited primordial follicle formation in offspring. **A** Average litter weight of PD3 mice in CTRL and SD groups (*n* = 5). **B** Ratio of ovarian weight to body weight in PD3 mice (*n* = 10). **C** Representative sections of CTRL and SD ovaries after MVH (red) and Hoechst (blue) nuclei staining. Scale bar = 100 and 50 μm. **D** Statistical results of cysts and follicles in PD3 ovaries in CTRL and SD groups (*n* = 6). **E**, **F** Representative WB images and relative expression levels of LHX8 and SOHLH1 proteins in PD3 ovaries of CTRL and SD groups (*n* = 5). **G** Principal component analysis (PCA) of the top 500 variable genes in the CTRL and SD groups. **H** Volcano map of gene transcription changes caused by SD. **I** Enrichment analysis of differential genes. **J** Heatmap of related genes in response to iron ions signaling pathway. **K** The relative expression of genes involved in response to iron ions (*n* = 5).

To comprehensively evaluate the impact of SD on ovarian development in offspring mice, we measured body weight and ovarian index on postnatal day 3 (PD3). Results showed that the average litter weight of mice in the SD group was significantly lower than that of CTRL (Fig. 3A). After calculating the ovarian index based on the ratio of ovarian weight to body weight, we found it to be markedly reduced after SD (Fig. 3B), indicating that maternal SD during pregnancy impaired ovarian development in offspring.

Furthermore, we stained sections of ovarian tissue from PD3 female mice. Compared to CTRL, results indicated that the proportion of follicles in the ovaries of SD mice was lower, suggesting that maternal SD during pregnancy may have had a detrimental effect on PF formation in offspring (Fig. 3C, D). In addition, we observed that the expression of key regulatory proteins for PF formation, such as LHX8 and SOHLH1, decreased in the ovaries of offspring from SD mothers (Fig. 3E, F). Further, we conducted RNA-seq with PD3 ovaries from CTRL and SD groups, and the molecular mechanisms of SD on germ cell loss in the offspring were investigated. The Principal component analysis (PCA) scatter plot confirmed significant changes within gene expression caused by SD (Fig. 3G). Based on results, 138 differentially expressed genes were identified; 75 genes were upregulated and 63 genes were downregulated in SD ovaries compared with those of CTRL (Fig. 3H). Notably, these differential genes were mainly enriched in biological processes, such as “response to iron ion”, “regulation of apoptotic process involved in morphogenesis”, and “negative regulation of angiogenesis” (Fig. 3I). Among them, the gene enrichment rate in the “response to iron ion” pathway was the highest, which indicated that it might play a dominant role in the effects of SD on ovarian cells. Additionally, of the three genes (*Tgfb2*, *Pax8*, and *Notch1*) involved in the “regulation of apoptotic process involved in morphogenesis”, two genes (*Pax8* and *Notch1*) were also identified in the “response to iron ion” pathway, indicating that there may be partial overlapping among these terms. Consequently, we further illustrated the changes of gene expression within the “response to iron ion” pathway (Fig. 3J), and mRNA quantification results of some genes were highly consistent with the RNA-seq data, thus validating the reliability of the transcriptome data (Fig. 3K).

### Maternal sleep deprivation during pregnancy induced ovarian cell ferroptosis in offspring

**Fig. 4:**
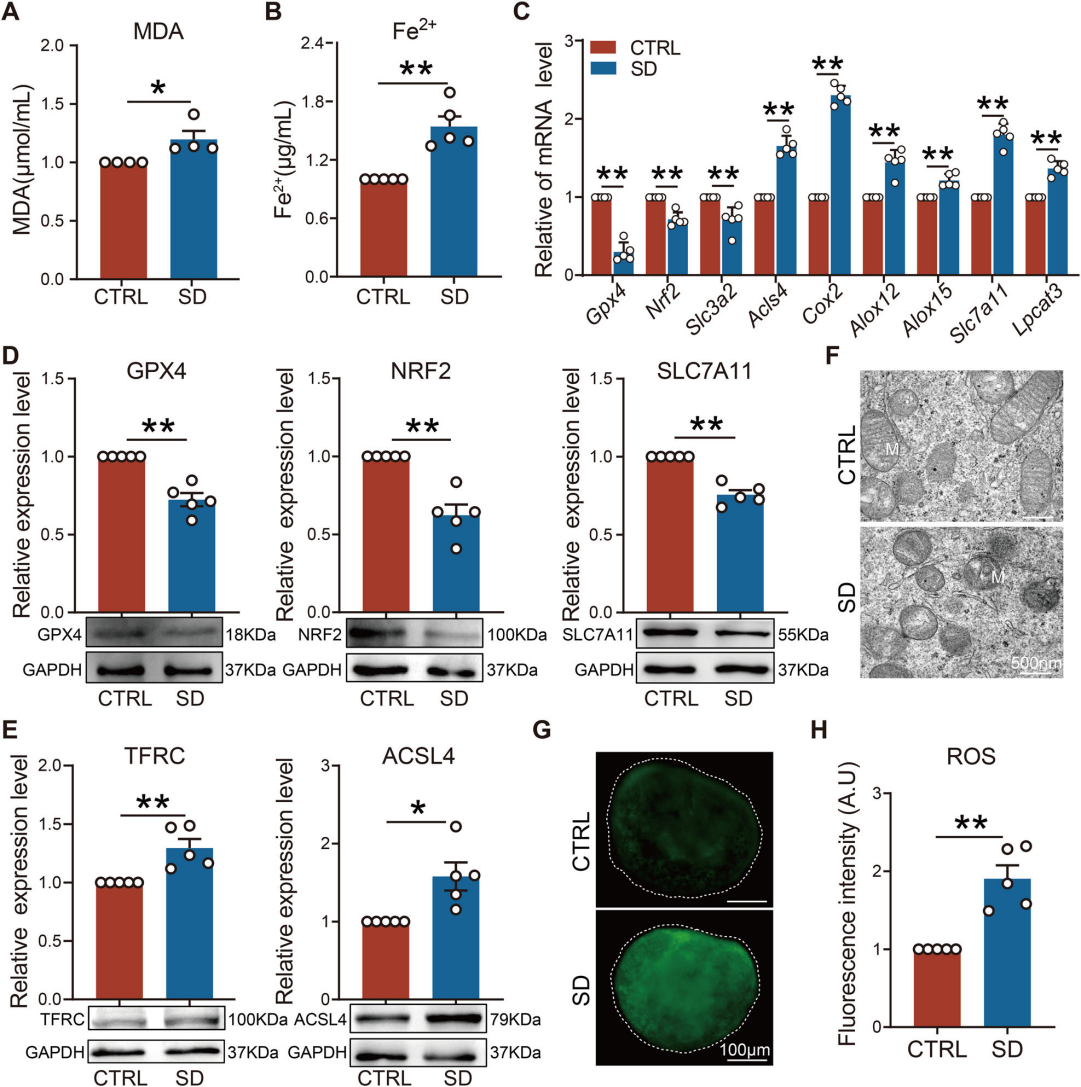
Maternal sleep deprivation during pregnancy induced ovarian ferroptosis in offspring. **A**, **B** The content of MDA (*n* = 4) and Fe^2+^ (*n* = 5) in the ovaries of PD3 in CTRL and SD groups. **C** mRNA expression levels of ferroptosis related genes in PD3 ovaries (*n* = 5). **D**, **E** Representative WB images and relative expression levels of GPX4, NRF2, ACSL4, SLC7A11, and TFRC proteins in PD3 ovaries of CTRL and SD groups (*n* = 5). **F** Transmission electron microscopy (TEM) images of mitochondrial morphologies in CTRL and SD groups. Scale bar = 500 nm. **G**, **H** Representative images and quantification of ROS fluorescence in ovaries of CTRL and SD groups (*n* = 5). Scale bar = 100 μm.

Transcriptomic results suggested that maternal SD might induce ferroptosis in the ovaries of offspring. Consequently, we measured malondialdehyde (MDA) levels in PD3 ovaries and found it to be elevated when compared to CTRL (Fig. 4A). At the same time, we detected Fe²⁺ levels in ovaries (the accumulation of which is a typical marker of ferroptosis) and these were also significantly upregulated in SD (Fig. 4B). In addition, expression levels of ferroptosis, including *Gpx4*, *Nrf2*, *Acsl4*, *Alox15*, and *Slc7a11*, were disrupted in SD (Fig. 4C). Meanwhile, WB results also revealed abnormal changes in the protein expression of GPX4, NRF2, SLC7A11, ACSL4, and TFRC (Fig. 4D, E). Notably, by using transmission electron microscopy (TEM), the increase of mitochondrial membrane density, shrunken volume, and a reduction of mitochondrial cristae in oocytes from the SD group were observed, all of which are typical characteristics of ferroptosis (Fig. 4F). Furthermore, the fluorescence intensity of reactive oxygen species (ROS) in SD ovaries was much higher than that of CTRL, indicating increased oxidative stress levels and an imbalance in the generation and degradation of ROS (Fig. 4G, H). Additionally, we examined ferroptosis markers in germ cells at distinct meiotic stages within the E16.5 genital ridges. Compared with CTRL, GPX4 expression in SD was reduced at the pachytene and diplotene stages, whereas ACSL4 expression showed an upward trend at zygotene, pachytene, and diplotene stages (Fig. S1). These results suggested that the developmental fate of germ cells in offspring with maternal SD may be closely related to the process of ferroptosis.

### Inhibition of ferroptosis effectively reduced germ cell loss in female offspring from sleep-deprived mice

**Fig. 5:**
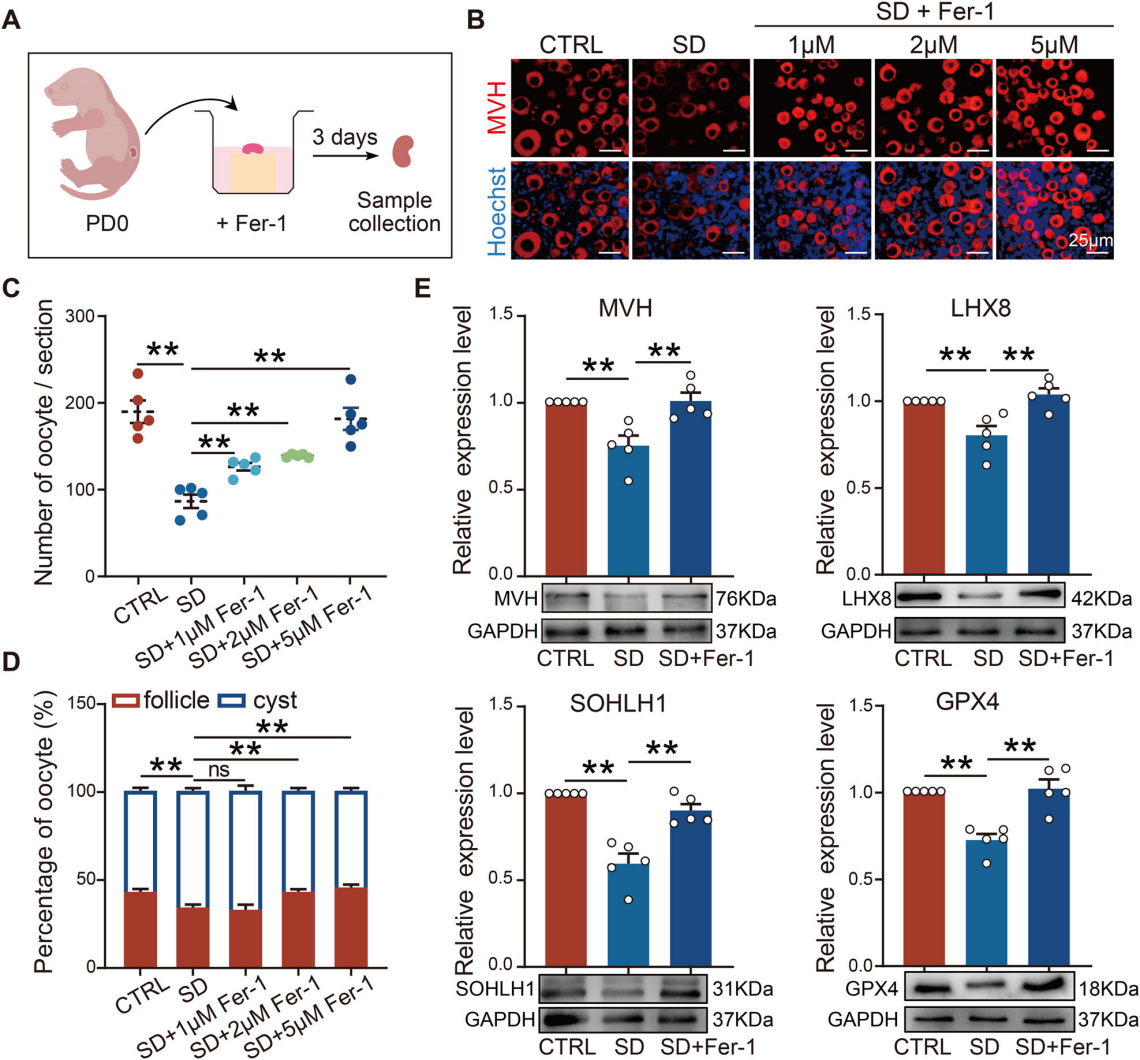
Inhibition of ferroptosis in vitro alleviated germ cell loss caused by maternal sleep deprivation during pregnancy. **A** Schematic diagram of in vitro ferroptosis inhibition. **B** Representative immunofluorescence images of MVH (red) staining in PD3 ovaries after in vitro inhibition of ferroptosis. Scale bar = 25 μm. **C**, **D** The number of germ cells and proportion of cysts and follicles in the ovaries after in vitro inhibition of ferroptosis (*n* = 5). **E** Representative WB images and relative expression levels of MVH, LHX8, SOHLH1, and GPX4 proteins in the ovaries (*n* = 5).

To gauge the involvement of ferroptosis in germ cell loss in SD, the ferroptosis inhibitor Ferrostatin-1 (Fer-1) was applied during in vitro culture of PD0 ovaries (Fig. 5A). Results indicated that inhibiting ferroptosis in the ovaries of sleep-deprived offspring could effectively mitigate germ cell loss; in particular, in the 5 μM Fer-1 group, the number of germ cells and the ratio of germ cells within follicles could be restored to near normal levels (Fig. 5B–D). Furthermore, the expression levels of key regulatory proteins for follicle formation, such as MVH, LHX8, and SOHLH1, were also correspondingly restored, as well as GPX4, a key regulator of ferroptosis (Fig. 5E). These findings suggest that ferroptosis may be an important factor leading to germ cell loss in female offspring of SD mothers.

**Fig. 6:**
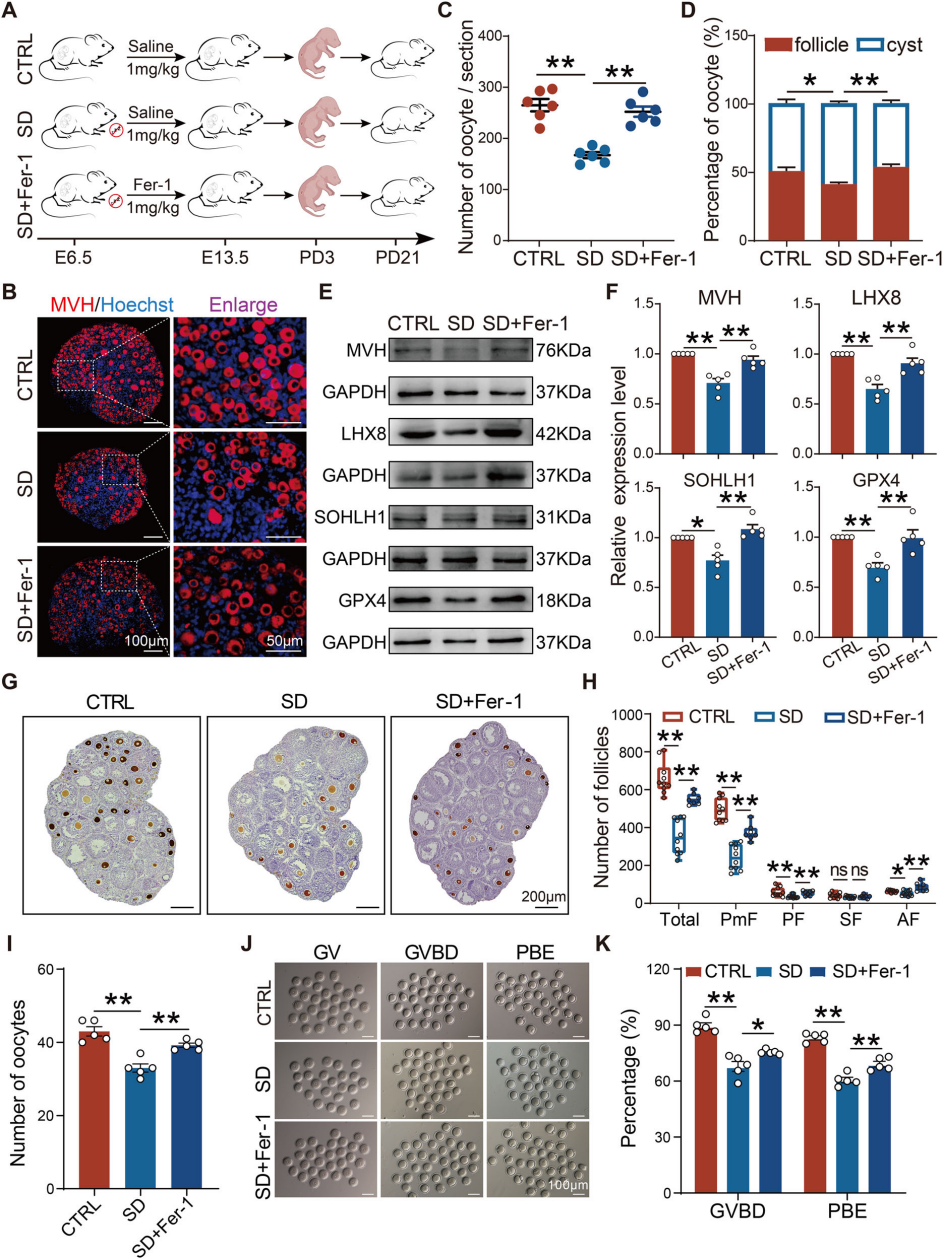
In vivo inhibition of ferroptosis alleviated germ cell loss induced by maternal sleep deprivation during pregnancy. **A** Schematic diagram of in vivo ferroptosis inhibition. **B** Representative immunofluorescence images of MVH (red) staining in PD3 ovaries after in vivo inhibition of ferroptosis. **C**, **D** The number of germ cells and proportion of cysts and follicles in the ovaries after in vivo inhibition of ferroptosis (*n* = 6). **E**, **F** Representative WB images and relative expression levels of MVH, LHX8, SOHLH1, and GPX4 proteins in the ovaries after in vivo inhibition of ferroptosis (*n* = 5). **G** Representative images of ovarian sections with immunohistochemistry of MVH-positive oocytes in PD21 ovaries. Scale bar = 200 μm. **H** Number of follicles of different classes in the ovaries of PD21 offspring (*n* > 7). **I** The number of oocytes obtained from ovaries of three groups (*n* = 5). **J** Representative images of oocyte at GV, GVBD, and PBE stages. Scale bar = 100 μm. **K** The percentages of oocytes reaching GVBD and PBE in each group (*n* = 5).

Further, we intraperitoneally injected 1 mg/kg of Fer-1 to SD pregnant mice (Fig. 6A). Consistent with the in vitro results, after the administration of Fer-1, the number of germ cells, and the ratio within follicles in PD3 ovaries were restored to levels similar to those of CTRL (Fig. 6B–D). WB analysis also showed that levels of MVH, LHX8, SOHLH1, and GPX4 proteins in the Fer-1 group were significantly higher than those of SD (Fig. 6E, F). These results further confirm the close relationship of ferroptosis with germ cell loss in offspring ovaries due to maternal SD during pregnancy.

To further assess the long-term impact of maternal SD on offspring fertility, the follicular development and oocyte developmental competence in offspring at PD21 were examined (Fig. 6A). Results showed that, compared with CTRL, the total number of follicles in ovaries from the SD group were significantly reduced, as well as the number of different stage follicles, particularly PFs, which were restored to a certain extent after inhibition of ferroptosis (Fig. 6G, H). To determine the effects of maternal SD on follicle function, we collected oocytes at GV stage for in vitro maturation. Results indicated that the number of oocytes obtained from the ovaries of offspring subjected to maternal SD was significantly decreased (Fig. 6I). Moreover, the number of oocytes reaching GVBD was relatively lower in offspring with maternal SD than that of CTRL; in addition, the first PBE rate of oocytes was markedly improved after ferroptosis inhibition (Fig. 6J, K). These findings suggest that the rescue effect of Fer-1 is not only reflected in the short-term restoration of germ cell numbers, but also supports the long-term developmental competence of follicles.

## Discussion

Changes in the maternal environment during pregnancy play a decisive role in fetal health. As a common stressor during pregnancy, SD can cause a series of physiological responses, such as disruption of the maternal endocrine system and an increase in oxidative stress levels [39]. We observed that maternal SD during pregnancy led to significant changes in serum hormone levels. E2 and PROG are key physiological components for implantation and maintenance of pregnancy [40, 41], the decline of which may result in insufficient nutritional and metabolic support for the fetus, and further impair the development and survival of fetal germ cells, ultimately leading to a reduction in germ cell number. As reported, high levels of maternal steroid hormones help maintain the integrity of ovarian cysts before birth; it is suggested that after parturition, falling estradiol triggers cyst breakdown and associated oocyte loss [42]. Furthermore, LH supports corpus luteum function during early pregnancy; a decrease of which may impair follicular development, affecting oocyte quality and quantity, and thereby influencing offspring germ cell reserve. An elevated FSH/LH ratio is recognized as a marker of diminished ovarian reserve and poor IVF outcomes, including a low number of follicles, oocytes, and embryos [43, 44]. Studies show that insufficient sleep during pregnancy can be associated with adverse pregnancy outcomes, including miscarriage and intrauterine growth restriction [45]. During the second trimester, SD negatively affects motor coordination development in offspring by activating inflammatory responses in the cerebellar vermis [46]. Although a substantial number of studies have demonstrated the effects of SD on mothers and offspring during pregnancy, the relationship between maternal SD and fetal reproductive health remains underexplored. In this study, we established an SD model during pregnancy in female mice to investigate the impacts of SD on ovarian development in female offspring and its potential mechanisms.

SD during pregnancy likely causes meiotic defects in germ cells in offspring. After sexual differentiation in mammals, the oogonia gradually enter meiosis and arrest at the diplotene stage of meiotic prophase I until ovulation [47]. Abnormalities in meiosis can lead to an inability to produce functional gametes or result in low-quality gametes, thereby causing issues such as infertility, spontaneous abortion, or congenital birth defects [48]. Our results revealed distinct changes in meiotic progression, including an increased length of the leptotene and zygotene stages; in addition, the process of DNA repair was affected. Moreover, we observed significant shifts in ferroptosis markers within germ cells during distinct meiotic stages at E16.5. Although massive germ-cell death had not yet been triggered, changes in GPX4 and ACSL4 indicated an elevated susceptibility to ferroptosis at this stage. We therefore propose that these alterations potentially constitute an “early-risk signature” that continues to intensify, driving ferroptosis-dependent germ-cell clearance at PD3, a conclusion further corroborated by Fer-1 rescue experiments. In addition, the decelerated meiotic progression likely caused germ cells to remain in critical developmental phases for an excessive period, thereby impairing proper PF assembly. Previous studies have shown that inhibiting the synaptonemal complex protein SYCP1 in rats leads to premature oocyte progression to the diplotene stage and accelerated PF assembly [49]. Similarly, suppression of cAMP signaling has been shown to delay meiotic prophase and reduce PF number [50]. Other studies have highlighted the essential role of meiosis-related genes in PF assembly [51]. For instance, conditional knockout of *Srsf1* in oocytes disrupted PF formation in mice, with meiotic defects identified as a primary cause of this abnormality [52]. Collectively, these findings underscore the close relationship between meiotic abnormalities and folliculogenesis. Similarly, studies have found that maternal obesity leads to defects in meiotic division and epigenetic alterations in embryonic oocytes, thereby affecting the future quality of fetal oocytes [53]. Furthermore, it has been suggested that meiotic defects may trigger an increase in germ cell genetic variations, as well as oocyte elimination during cyst breakdown, and have long-term effects on the health and development of offspring [36]. Based on the above results, abnormal meiotic development in germ cells may lead to adverse pregnancy outcomes, such as abnormal primordial follicle assembly, germ cell loss, and an increased abortion rate. Additionally, studies report that insufficient sleep duration in pregnant women is associated with an increased risk of low birth weight in their offspring [54]. Consistent with our results, SD during pregnancy reduced body weight and ovarian index in mouse offspring.

Maternal SD during pregnancy diminished offspring ovarian reserve, and the massive germ cell loss caused by SD may be associated with ferroptosis. Currently, numerous studies recognize that SD can lead to the accumulation of a large amount of iron ions and lipid peroxidation, thereby inducing ferroptosis in mammalian cells [55, 56]. Studies have found that SD might cause ferroptosis in intestinal cells, particularly goblet cells [56]. Furthermore, during pregnancy, SD may also trigger ferroptosis in the hippocampal region of offspring rats [57]. These research findings shed light on the potentially important roles of ferroptosis in related diseases caused by SD. Our research found an increase in ROS, Fe^2+^, and MDA levels in the ovaries of offspring from SD pregnancies, along with mitochondrial atrophy, disappearance of cristae, as well as the increased expression of proteins associated with ferroptosis, all of which support the role of ferroptosis in the loss of germ cells in offspring due to maternal SD. In our transcriptomic analysis, multiple biological processes were enriched, indicating that maternal SD might have affected germ cell development through various mechanisms, including the activation of apoptotic pathways, inhibition of angiogenesis, and induction of ferroptosis, with ferroptosis potentially playing a dominant role. Alterations in the “response to iron ion” signaling pathway may lead to abnormal intracellular iron metabolism, resulting in elevated levels of free Fe²⁺. When intracellular iron levels rise, iron reacts with lipid peroxides, triggering a chain reaction of lipid peroxidation and further exacerbating ferroptosis [58]. Additionally, changes in the “response to iron ion” pathway might affect the antioxidant capacity of cells, particularly the activity of GPX4, thereby reducing cellular antioxidant defense [59]. Based on the above analysis, we hypothesized that during maternal SD, iron overload could intensify oxidative stress, exacerbate lipid peroxidation, and result in ferroptosis; thus, dysregulation of iron metabolism might serve as the initial trigger of ferroptosis. A substantial number of researchers have recognized that ferroptosis is closely related to reproductive health, and various factors may affect iron metabolism and oxidative stress status, which then go on to affect the function of germ cells and fertility. Studies indicate that iron overload in follicular fluid can trigger ferroptosis in granulosa cells and impair oocyte maturation, thereby increasing the risk of infertility associated with endometriosis [60]. As reported, BNC1 is a transcription factor involved in oocyte and follicle generation, and its deficiency in oocytes induces oocyte ferroptosis, ultimately leading to oocyte death and follicle atresia [61]. Additionally, ferroptosis also plays a significant role in the onset and progression of female ovarian dysfunction, and it may induce ovarian dysfunction, damage pre-implantation embryos, reduce endometrial receptivity, and increase the crosstalk between diseases associated with subfertility [62, 63]. Ferrostatin-1 (Fer-1) is a specific small molecule inhibitor of ferroptosis; as a potent antioxidant, it can scavenge lipid hydroperoxides in the presence of ferrous iron to suppress ferroptosis [64]. Studies have shown that cisplatin treatment leads to iron ion accumulation, thereby inducing ferroptosis in ovarian tissues, and the use of Fer-1 can inhibit cisplatin-induced ovarian damage and granulosa cell death [65]. Additionally, treatment with Fer-1, both in vivo and in vitro, was found to restore the significant loss of germ cells caused by SD in the study, further confirming the involvement of ferroptosis in massive germ cell loss. These findings provide a new perspective in understanding the molecular mechanisms by which SD during pregnancy affects reproductive health in offspring.

Importantly, our results implied that maternal sleep disorder may be another factor impacting premature ovarian insufficiency (POI) in offspring. PF assembly is an important event in early ovarian development; it occurs during mid-term in humans and perinatal in rodents. It is understood that the mother is extremely sensitive to external stimulation during pregnancy, especially the critical window period of fetal ovarian development, including PF formation [18]. An impaired PF assembly process may lead to a reduction in PF numbers, increasing the risk of diseases such as POI, which can severely affect female fertility [12]. Similarly, perinatal maternal exposure to the environmental endocrine disruptor bisphenol S accelerates the breakdown of germ cell cysts and impacts the progress of PF assembly in neonates, leading to excessive oocyte loss [66]. Furthermore, research has discovered that maternal factors during pregnancy, such as nutritional status, can impact PF formation and ovarian reserve in offspring [67]. In addition, some clinical investigations show that chronic and cumulative adverse life events have a relationship with the onset of POI in young women [68]. A recent report indicates that SD can promote excessive PF activation in a β2 adrenergic receptor-dependent manner, causing POI in mice [27]. Moreover, our previous study suggests that gut dysbiosis mediated systemic metabolomics could reduce ovarian reserve and impact female fertility in adolescent mice [28]. In this study, an examination of follicular development and oocyte quality in PD21 offspring from sleep-deprived mothers shows a significantly decreased ovarian reserve. Thus, beside the genetic defects, autoimmune damage, and environmental pollutants, the study suggests that maternal sleep quality may be another factor in POI in offspring.

Collectively, our findings indicated that maternal SD during pregnancy may have profound effects on their offspring’s ovarian function. We found that maternal SD during gestation disrupted normal meiotic progression and primordial follicle assembly, leading to a massive loss of germ cells. Importantly, our study also found that inhibiting ferroptosis significantly mitigated the negative impact of maternal SD on the number of germ cells in offspring. These findings enhance our understanding of the mechanisms by which maternal sleep disorders during pregnancy affect the reproductive health of offspring.

## Materials and methods

### Animal treatment and sample collection

Experimental CD1 mice were purchased from Jinan Experimental Animal Breeding Center (Jinan, China). Mice were housed in an experimental animal room (temperature 23–24°C, humidity 50%) under a 12-h alternating cycle of light and darkness, with ample food and water. Seven-week-old female mice with swollen vaginal orifices and exhibiting estrus were selected for mating with male mice after 6 p.m. The presence of a vaginal plug the next day was recorded as embryonic stage 0.5 (E0.5). Pregnant mice at E6.5 were divided into a normal control group (CTRL) and a sleep deprivation group (SD). A mouse SD model was established using a widely validated SD device (KW-BD, NJKEW Biotechnology Co., Ltd., Nanjing, China) [28, 69–71], which was applied to pregnant mice for 18 h/day. The SD device operated at a speed of 1.0 r/min, with a single running cycle of 24 h. After 7 days of SD, ovarian tissues from offspring mice at E16.5, E18.5, and after birth at PD0, and PD3 were collected for subsequent experiments.

To investigate the protective effect of ferrostatin-1 (Fer-1, SML0583; Sigma, USA) on reproductive damage in offspring caused by maternal SD during pregnancy, mice at E6.5 were divided into three groups: the CTRL group (intraperitoneal injection of saline), the SD group (SD with concurrent intraperitoneal injection of saline), and the SD + Fer-1 group (SD with concurrent intraperitoneal injection of Fer-1, according to a previous study, 1 mg/kg Fer-1 was used as the working concentration) [55]. All mouse experimental procedures were approved by the Animal Ethics Committee of Qingdao Agricultural University (approval number: 2022-0021).

### Immunohistofluorescence (IHF)

After overnight fixation with 4% paraformaldehyde and flushing, the ovarian tissue was dehydrated with gradient alcohol and paraffin embedded. The embedded ovarian tissues were sectioned at 5 μm and the sections were placed on slides. After rehydration through graded alcohol and antigen repair, the slides were blocked for 45 min and then incubated with the primary antibody overnight at 4 °C. Next day, the slides were incubated with the secondary antibody at 37 °C in the dark for 45 min and stained with Hoechst33342 (Beyotime, C1022, Shanghai, China) for 5 min to reveal cell nuclei. Finally, the slides were mounted with an anti-fluorescence attenuating tablet (AR1109, BOSTER, Wuhan, China) and observed under a Olympus BX51 microscope for photography.

### Histology and immunohistochemistry (IHC)

The detailed procedure of ovarian immunohistochemistry (IHC) has been described in previous articles [28]. Briefly, ovarian sections were treated with xylene for 30 min and then rehydrated with graded alcohol. Antigen retrieval was performed in citrate buffer at 96°C for 10 min, followed by cooling and sequential rinsing in Tris-buffered saline (TBS) and TBST (TBS with Tween 20) for 5 min each. Subsequently, the samples were incubated with 3% H_2_O_2_ to remove endogenous peroxidase activity and blocked with BDT (TBS containing 3% BSA and 10% goat serum) for 30 min, followed by overnight incubation with primary antibodies at 4 °C. Next day, slides were incubated with a peroxidase-conjugated secondary antibody (Beyotime, A0208, Shanghai, China) at 37 °C for 45 min. Finally, samples were stained using a DAB kit (zsbb-bio, ZLI-9017, Beijing, China) and counterstained with hematoxylin (Sangon Biotech, E607317). After sealing with neutral resin, the slides were examined under a microscope.

### Western blot (WB)

An appropriate amount of RIPA lysis buffer (Beyotime, P0013C, Shanghai, China) was added to ovarian tissue samples; the samples were thoroughly lysed before the addition of sodium dodecyl sulfate (SDS) buffer, and proteins were denatured by boiling in water for 5 min. Electrophoresis was performed at a constant voltage of 120 V, and the proteins were transferred onto PVDF membranes (Millipore, ISEQ00010, USA) using the wet transfer method. The membrane was blocked with TBST containing 5% BSA for 4 h, followed by incubation with the primary antibody overnight at 4 °C. The next morning, after washing with TBST, the membrane was incubated with horseradish peroxidase-conjugated secondary antibody at room temperature for 90 min. The target proteins were detected using the BeyoECL Plus detection kit (Beyotime, P0018, Shanghai, China), with GAPDH serving as an internal control and the target proteins were analyzed using ImageJ software (1.48 v). Information regarding the antibodies used in this paper is listed in Table S1.

### Immunostaining of synaptonemal complexes

As published previously [72], genital ridges from E16.5 embryos were collected and subjected to hypotonic treatment with hypo extraction buffer (HEB); subsequently, 0.1 M sucrose was dropped onto a coverslip and the genital ridges were teased apart to release the cells into the sucrose, and then fixed with paraformaldehyde. The next day, immunofluorescence staining with a specific primary antibody of SYCP3 was performed. Finally, the chromosome structure was observed under a fluorescence microscope.

### ELISA

Following the manufacturer’s instructions, specific enzyme-linked immunosorbent assay (ELISA) kits were used to detect the levels of luteinizing hormone (LH) (JM-11607M2, Jingmei Biological Technology, Jiangsu, China), estradiol (E2) (JM-02849M2, Jingmei Biological Technology, Jiangsu, China), follicle stimulating hormone (FSH) (JM-02838M2, Jingmei Biological Technology, Jiangsu, China), and progesterone (PROG) (JM-02851M2, Jingmei Biological Technology, Jiangsu, China) in the serum of E13.5 pregnant mice. Concurrently, the kits were used to measure the content of Fe^2+^ (ADS-W-QT027, Aidisheng Biological Technology, Jiangsu, China) and MDA (ADS-W-YH002, Aidisheng Biological Technology, Jiangsu, China) in the ovaries of PD3 mice.

### Real‑time quantitative PCR (RT‑qPCR)

Total RNA of ovarian tissues was extracted according to the instructions of a SPARKeasy Improved Tissue/Cell RNA Extraction Kit (Sparkjade, AC0202, Shandong, China), and cDNA was generated through reverse transcription by using SPARKscript II RT Plus (Sparkjade, AG0304, Shandong, China). Real-time quantitative PCR (RT-qPCR) was performed using SYBR Premix Ex Taq II fluorescent dye and a CFX96 Real-Time PCR Detection System (BioRad-CFX96, USA). The primers used in this experiment are shown in Table S2.

### Detection of ROS level

Fresh ovarian tissue was placed in ROS staining solution (Beyotime, S0033S, Shanghai, China) and stained in an incubator at 37 °C for 25 min; they were subsequently washed twice. The ovaries were then photographed under a microscope on a slide.

### The in vitro culture of oocytes

Oocytes were cultured following standard protocols as described in previous papers [28]. In brief, ovaries were minced, the naked oocytes [germinal vesicle (GV) stage] were collected under the microscope and washed three times in M2 medium (Sigma, M7167). Subsequently, the oocytes were transferred to M16 droplets (Sigma, M7292) balanced for 3 h and cultured in an incubator with 5% CO_2_ at 37 °C. The number of oocytes undergoing germinal vesicle break-down (GVBD) and first polar body extrusion (PBE) was counted after 4–6 h and 12–16 h, respectively.

### RNA-seq sequencing

Total RNA was extracted from the ovaries according to the manufacturer’s instructions. The cDNA library was constructed with high-quality RNA by Annoroad Gene Technology Corporation (Beijing, China) and sequenced with an Illumina NovaSeq 6000 instrument using a 150 bp paired-end strategy. Based on the transcriptome sequencing results returned by the company, FastQC (v0.11.8) was used to process the data for quality control, and STAR (v2.7.0 f) software was applied for read alignment of high-quality data to the reference genome sequence. FPKM (fragment per kilobase of transcript per million mapped reads) values were calculated to quantify gene expression abundance. DESeq2 (1.42.1) software was used for differential expression analysis between different groups, and Metascape (v3.5) was used for pathway enrichment analysis.

### In vitro culture of ovaries

Newborn mouse ovaries were cultured in 24-well plates, with 400 μL of culture medium added to each well; all media were changed daily. The culture medium consisted of Dulbecco’s Modified Eagle Medium/F12 (DMEM/F12, Hyclone, SH30023.01B, USA) and α-Minimum Essential Medium (α-MEM, Hyclone, SH30265.01B, USA), supplemented with 0.23 mmol/L sodium pyruvate (Hyclone, SH40003-12, USA), 10% Fetal Bovine Serum (FBS; Gibco, 10099-141, USA), 100 IU/mL penicillin G, and 100 mg/mL streptomycin sulfate.

### Statistical analysis

All experiments contained at least three independent replicates. Data were processed using GraphPad Prism 8 software (GraphPad software Inc., San Diego, CA, USA), and differences between groups were analyzed by unpaired Student’s *t* test or one-way analysis of variance (ANOVA). Before performing an unpaired Student’s *t* test or one-way ANOVA, the normality and homogeneity of data were confirmed. Results are expressed as mean ± standard error of the mean (SEM). *P < 0.05 and **P < 0.01 were considered to indicate significant and highly significant differences, respectively.

## Supplementary information

Supplementary data

Original western blots

## Data Availability

The datasets used and/or analyzed during the current study are available from the corresponding author.
