# Supplementary material for: Maternal sleep deprivation during pregnancy induced offspring germ cells loss through ferroptosis
Source: Cell Death Discov. 2025 Nov 24;11:544. doi: 10.1038/s41420-025-02839-5 (PMC12644548; doi:10.1038/s41420-025-02839-5)
Supplement: Supplementary file 1 — Supplementary data [file 41420_2025_2839_MOESM1_ESM.docx]

**Maternal sleep deprivation during pregnancy induced offspring germ cells loss through ferroptosis**

Qingchun Liu ^1,#^, Jiamao Yan ^1,2,#^, Han Wang ^1,#^,Tao Wang ^1^, Kai Zhang ^3^, Aiying Li ^3^, Junjie Wang ^1^, Teng Zhang ^2^, Wei Shen ^1,*^, Lan Li ^1,*^

**Author information**

^#^ These authors contributed equally: Qingchun Liu, Jiamao Yan, Han Wang.

Authors and Affiliations

**College of Animal Science and Technology, Qingdao Agricultural University, Qingdao, 266109, China**

Qingchun Liu, Jiamao Yan, Han Wang, Tao Wang, Junjie Wang, Wei Shen & Lan Li

**State Key Laboratory of Reproductive Regulation and Breeding of Grassland Livestock (R2BGL), College of Life Sciences, Inner Mongolia University, Hohhot, 010070, China**

Jiamao Yan, Teng Zhang

**College of Life Sciences, Qingdao Agricultural University, Qingdao, China**

Kai Zhang, Aiying Li

Corresponding authors

* Correspondence to Wei Shen and Lan Li.

Correspondence and reprint requests to:

Prof. Wei Shen; E-mail: wshen@qau.edu.cn

Prof. Lan Li; E-mail: lli@qau.edu.cn

**Supplementary figures and figure legends：**


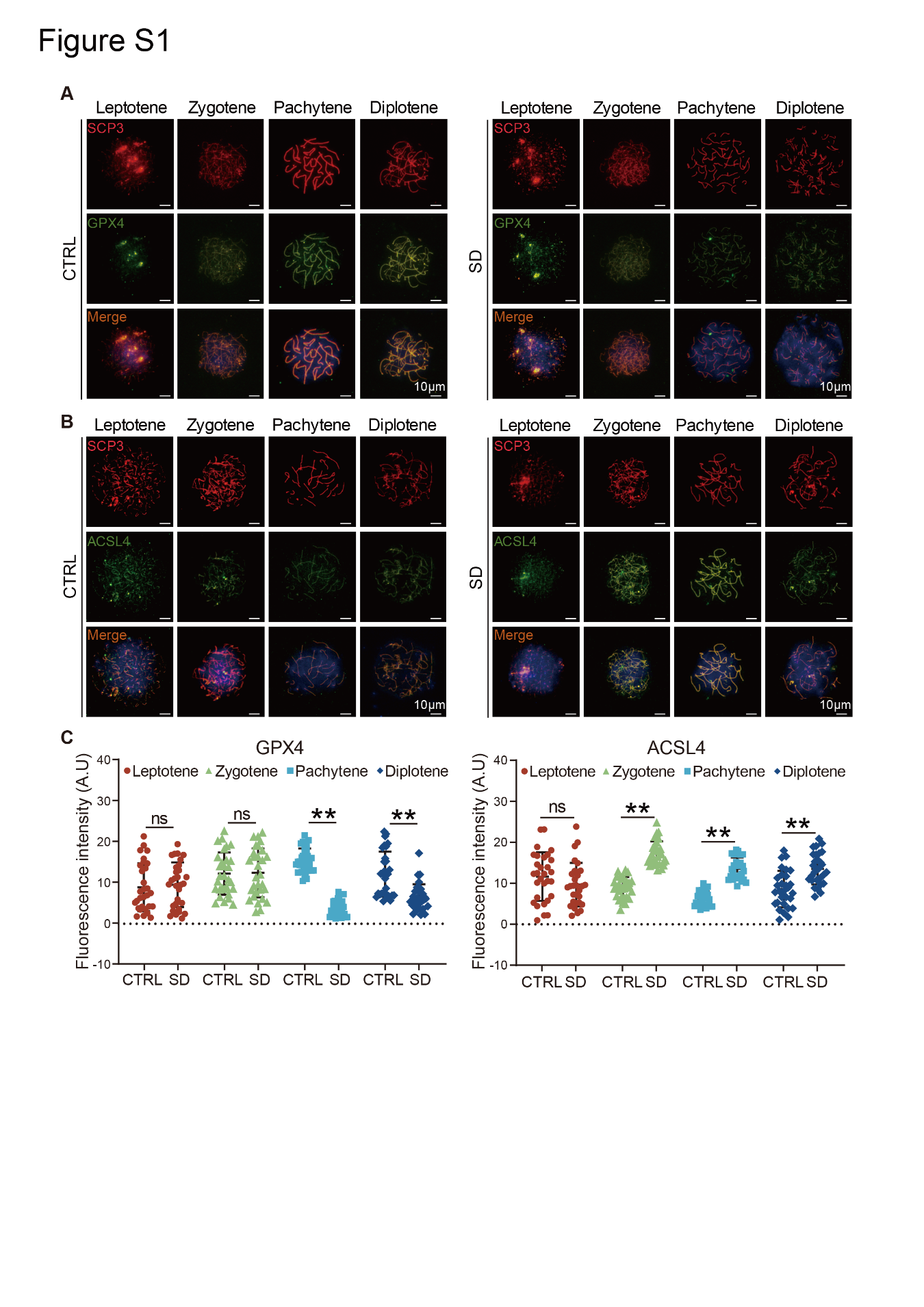


**Fig. S1 Effects of maternal sleep deprivation on the expression of ferroptosis markers in fetal germ cells at different meiotic stages.**

**A,B** Immunofluorescence staining of SCP3 (red), Hoechst (blue), and GPX4 (green, A) or ACSL4 (green, B) in oocytes at each meiotic stage with E16.5 ovaries. Scale bar = 10 µm. **C** Quantification of relative fluorescence intensities of GPX4 and ACSL4 proteins in oocytes at each meiotic stage (n = 30).

**Supplementary Table 1. Antibody information used in the study.**

| **Primary antibodies** | **Manufacturer and Product code** | | **Dilution** | | **Source** |
| --- | --- | --- | --- | --- | --- |
| MVH | Abcam（ab13840） | | WB 1:1000 IF 1:200 | | Rabbit |
| RAD51 | Abcam（ab133534） | | WB 1:1000 | | Rabbit |
| SCP3 | Abcam（ab97672） | | WB 1:1000 SC 1:200 | | Mouse |
| MLH1 | Abcam (ab92312) | | WB 1:1000 | | Rabbit |
| γ-H2AX | Abcam (ab26350) | | WB 1:1000 | | Rabbit |
| LHX8 | Abcam（ab137036） | | WB 1:1000 | | Rabbit |
| SOHLH1 | Biorbyt（orb158448） | | WB 1:1000 | | Rabbit |
| NRF2 | Abclonal（A21176） | | WB 1:1000 | | Rabbit |
| SLC7A11 | Abclonal（A2413） | | WB 1:1000 | | Rabbit |
| TFRC | Abclonal (A5865) | | WB 1:1000 | | Rabbit |
| GPX4 | Abclonal (A1933) | | WB 1:1000 SC 1:200 | | Rabbit |
| ACSL4 | Abclonal (A6826) | | WB 1:1000 SC 1:200 | | Rabbit |
| GAPDH | Affinity (AF7021) | | WB 1:2000 | | Rabbit |
| **Secondary antibodies** | | **Manufacturer and Product code** | | **Dilution** | **Source** |
| Donkey anti-mouse lgG H&L (Alexa Fluor® 555) | | Abcam（ab150106） | | 1:200 | Donkey |
| Donkey anti-rabbit lgG H&L  （AlexaFluor®555 | | Abcam（ab150074） | | 1:200 | Donkey |
| HRP-conjugated goat anti- rabbit IgG (WB) | | Beyotime (A0208) | | 1:1000 | Goat |
| HRP-conjugated goat anti- Mouse IgG (WB) | | Beyotime (A0216) | | 1:1000 | Goat |

**Supplementary Table 2. Primers used for quantitative RT-PCR.**

| Gapdh-F | GTCATTGAGAGCAATGCCAG |
| --- | --- |
| Gapdh-R | GTGTTGCTACCCCCAATGTG |
| Gpx4-F | AAGTCCTAGGAAACGCCCG |
| Gpx4-R | CATCGCGGGATGCACACAAG |
| Slc3a2-F | GGACCTCACTCCCAACTACC |
| Slc3a2-R | ACAAGGGTGCATTCATCAGC |
| Slc7a11-F | GCTCGTAATACGCCCTGGAG |
| Slc7a11-R | GGAAAATCTGGATCCGGGCA |
| Alox12-F | ATGAGATGCCTCCCAGGACT |
| Alox12-R | CACCTGTGCTCACTACCTGA |
| Lpcat3-F | ACTGAAGCTAATTGGGCTGTGT |
| Lpcat3-R | TCCAGCAATGAAGGGACACC |
| Cox2-F | CACACTCTATCACTGGCACC |
| Cox2-R | TCCAGGAGGATGGAGTTGTT |
| Nrf2-F | GACTACAGTCCCAGCAGAGTG |
| Nrf2-R | TCTGCGTGCTCAGAAACCTC |
| Alox15-F | CGGGGATGGAGAAGCTACAG |
| Alox15-R | GGGCAGTTCGAGCTGGATG |
| Acsl4-F | GTCCTTCGGTCCTAGTCCAG |
| Acsl4-R | GTCCTTCGGTCCTAGTCCAG |
| Tfr2-F | CCACAGAGTCTCTACCCCCA |
| Tfr2-R | CGAGGTCTGAACCCATTGCT |
| Sfrp1-F | CCTCTAAGCCCCAAGGTACA |
| Sfrp1-R | GCTCCTTCTTCTTGATGGGC |
| Pax8-F | AGCATTACCCGGAGGCCTAT |
| Pax8-R | AAGGGTGAATGAGGATCTGCC |
| Mt1-F | AGATCTCGGAATGGACCCCA |
| Mt1-R | AGGAGCAGCAGCTCTTCTTG |
| Gata1-F | GATGAATGGTCAGAACCGGC |
| Gata1-R | GTGGGCGGTTCACCTGAT |
| Notch1-F | GAAGGCTTCAGTGGCCCTAAT |
| Notch1-R | TGCATACCCCGCTGTTTTTG |
