# Supplementary material for: Maternal sleep deprivation during pregnancy induced offspring germ cells loss through ferroptosis
Source: Cell Death Discov. 2025 Nov 24;11:544. doi: 10.1038/s41420-025-02839-5 (PMC12644548; doi:10.1038/s41420-025-02839-5)

Original images for Figure 1

Fig. 1F

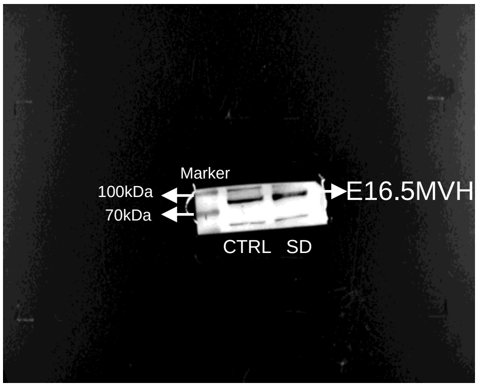

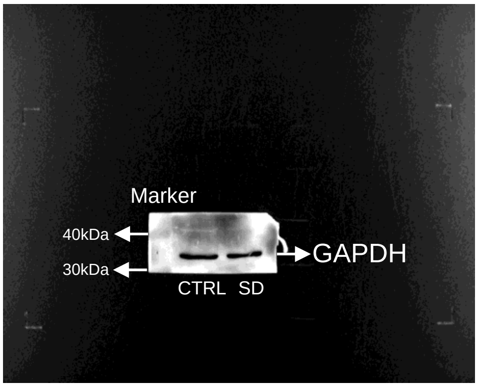

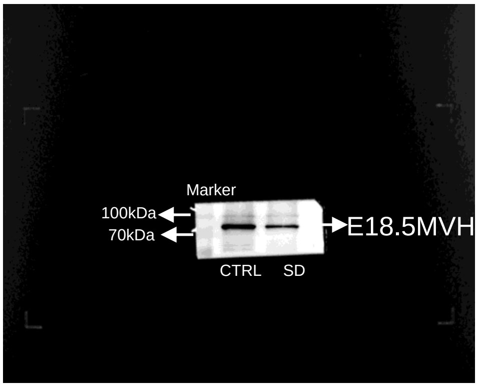

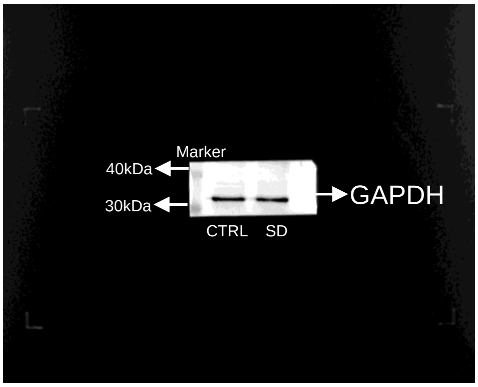

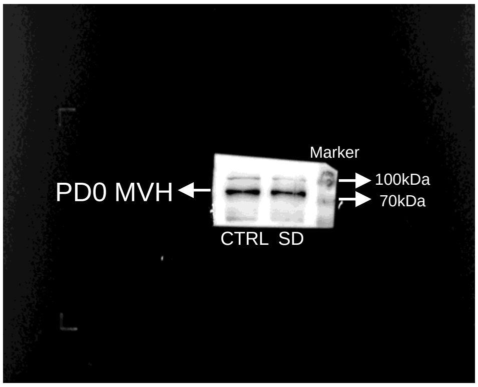

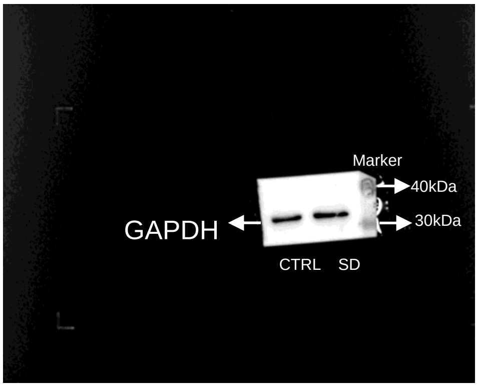

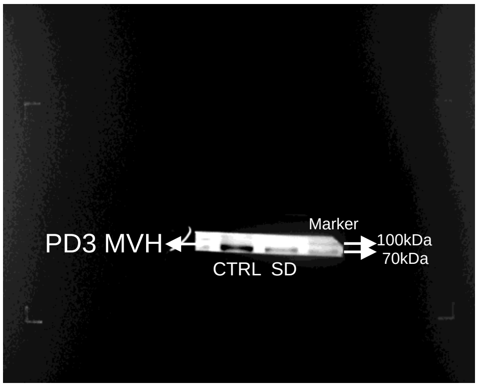

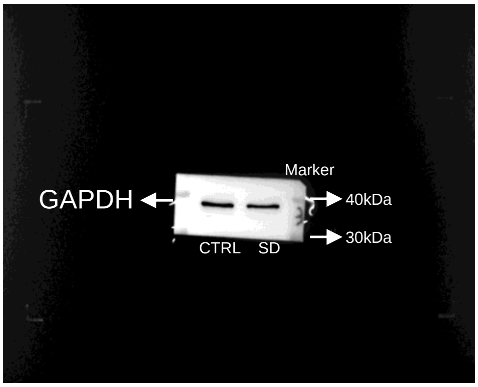

Original images for Figure 2

Fig. 2C

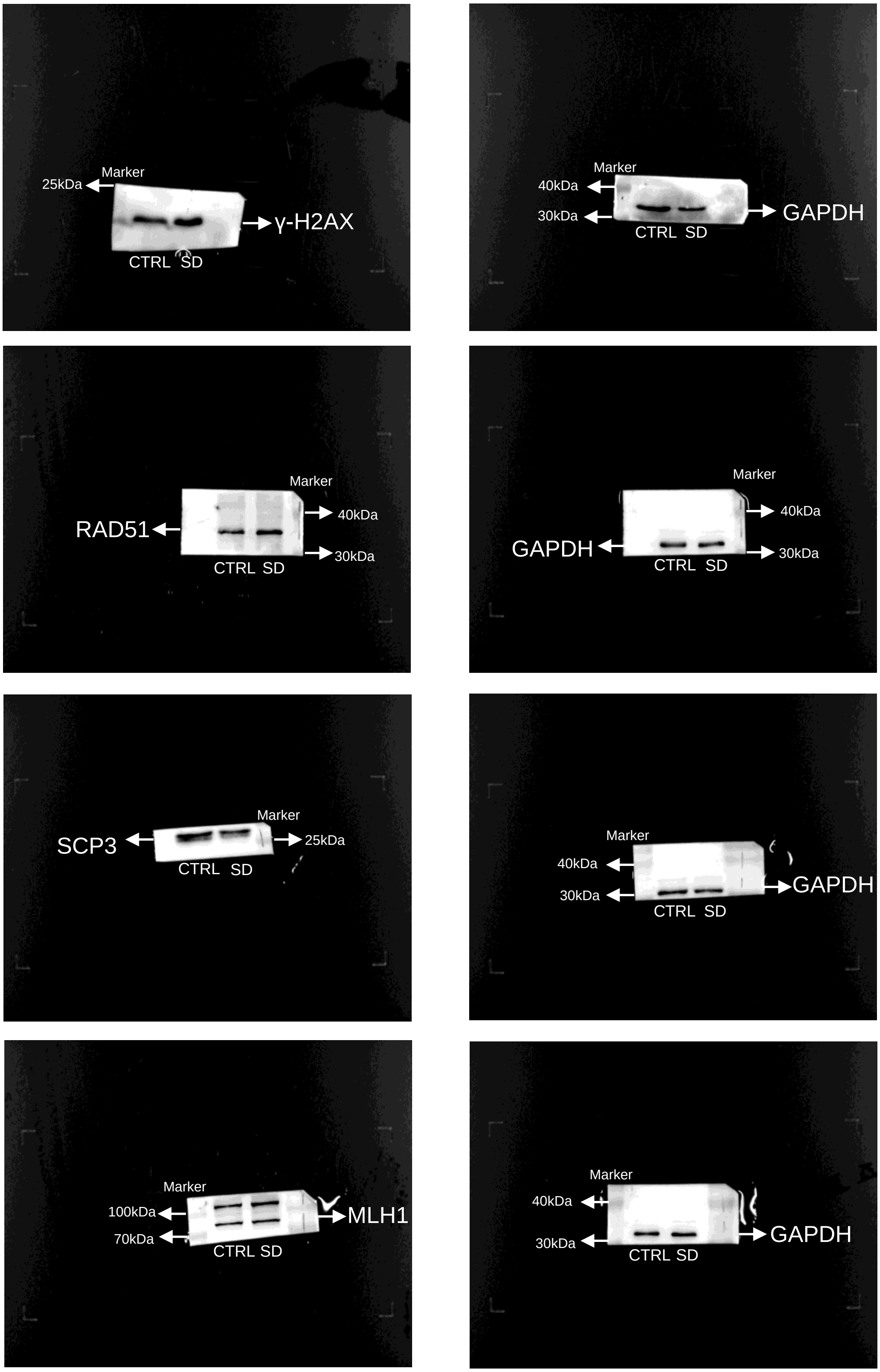

Original images for Figure 3

Fig. 3E,F

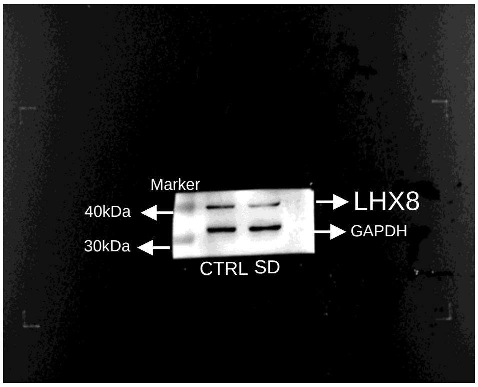

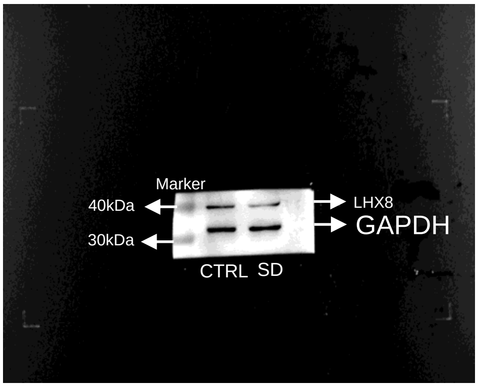

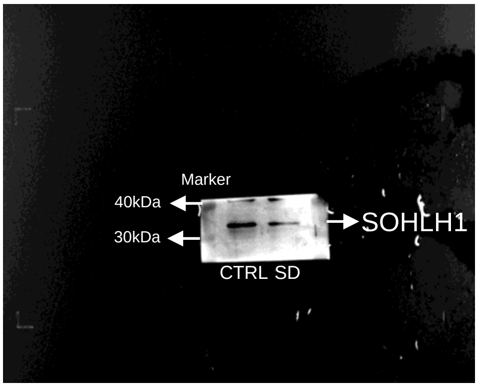

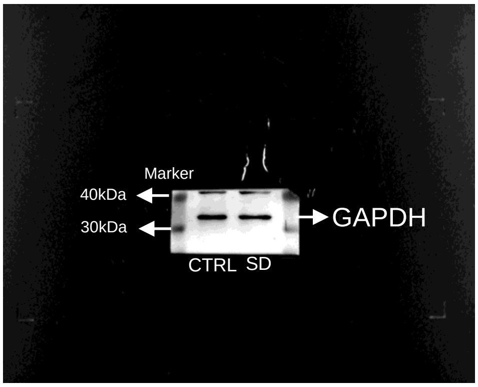

Original images for Figure 4

Fig. 4D

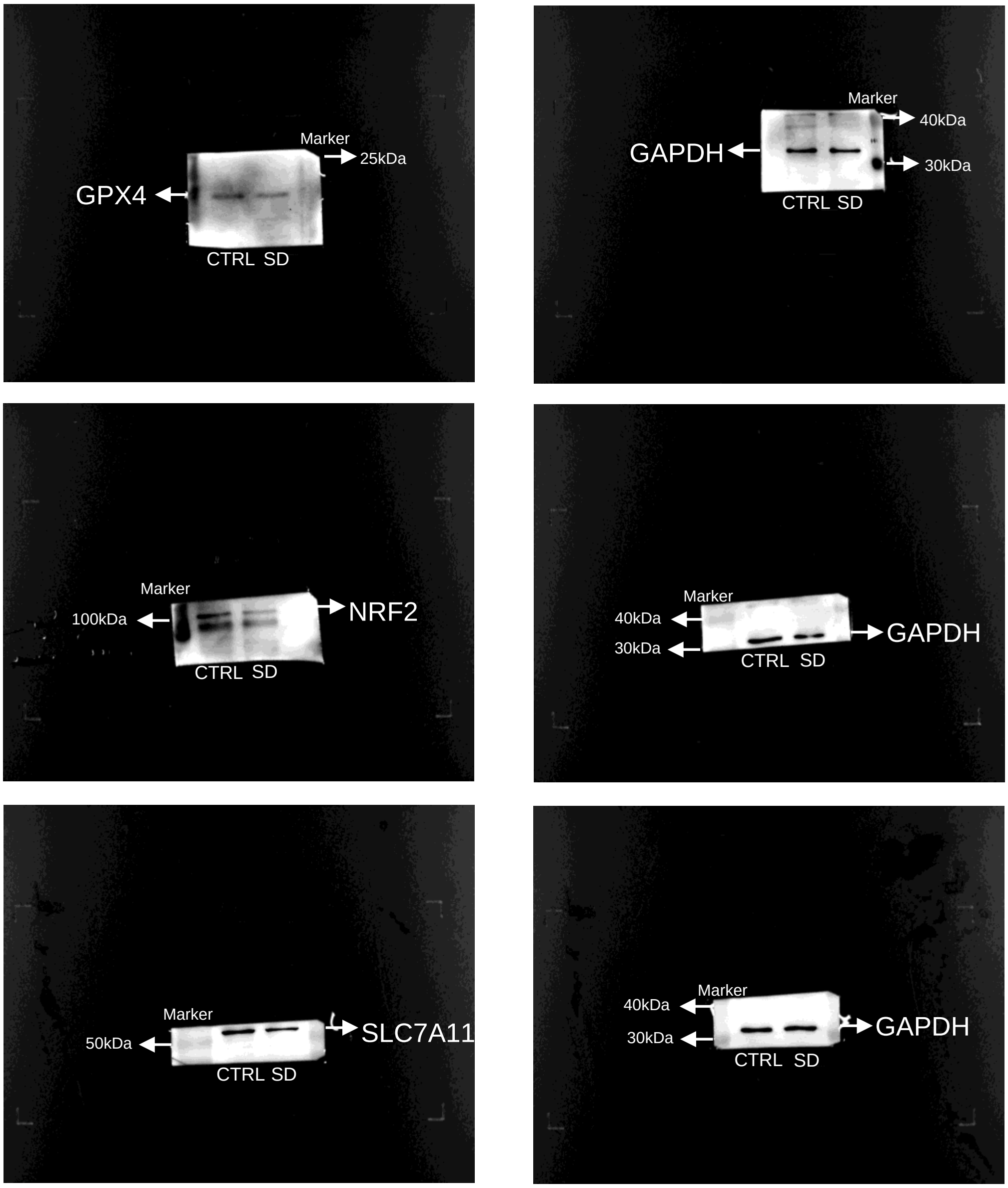

Original images for Figure 4

Fig. 4E

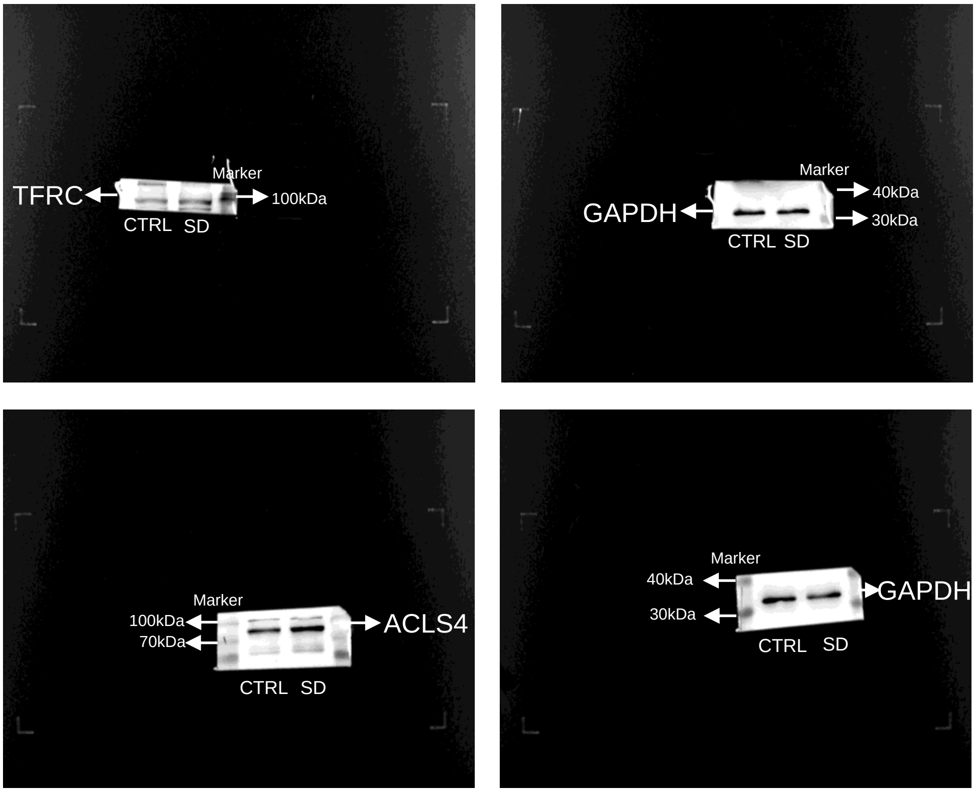

Original images for Figure 5

Fig. 5E

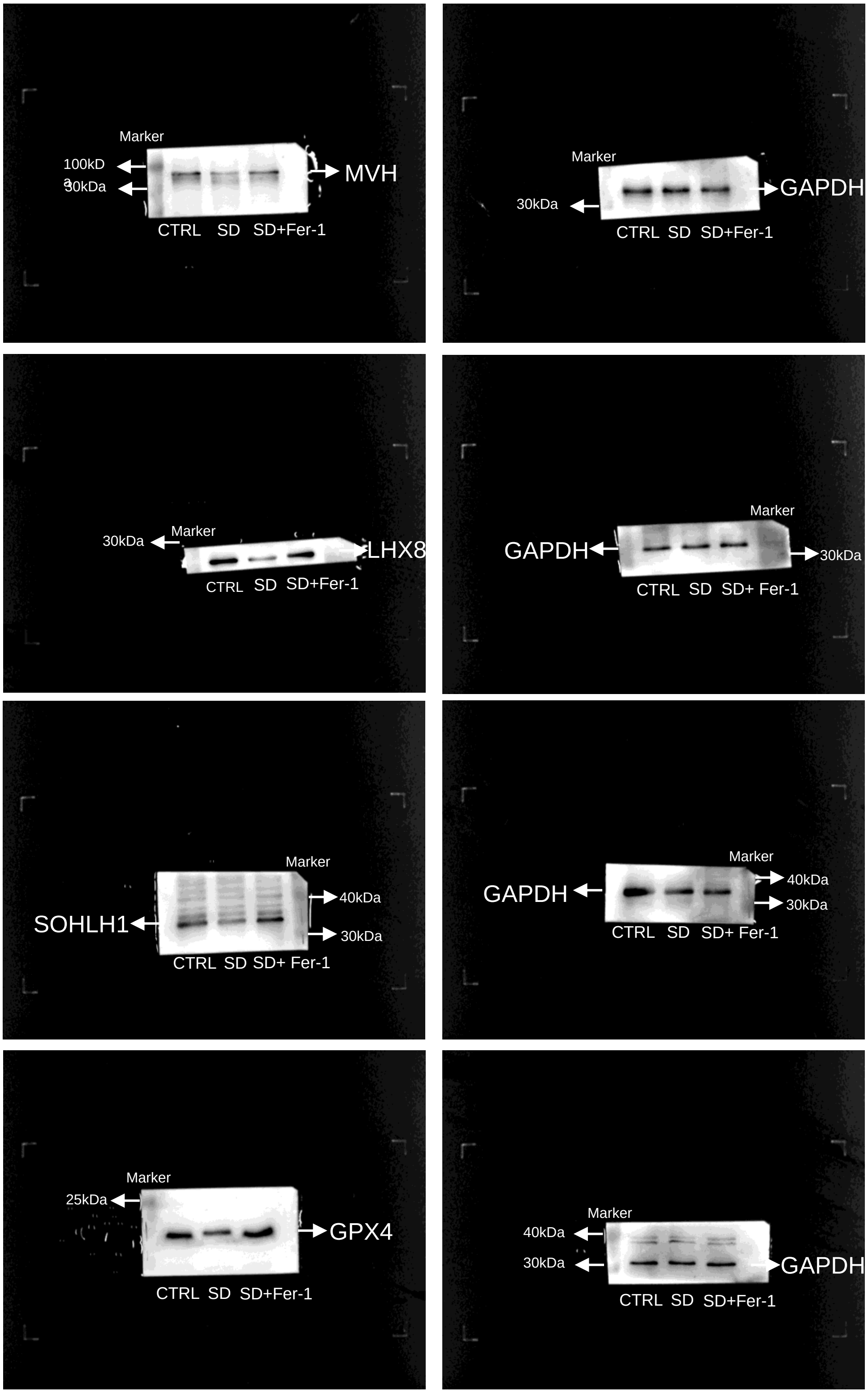

Original images for Figure 6

Fig. 6E

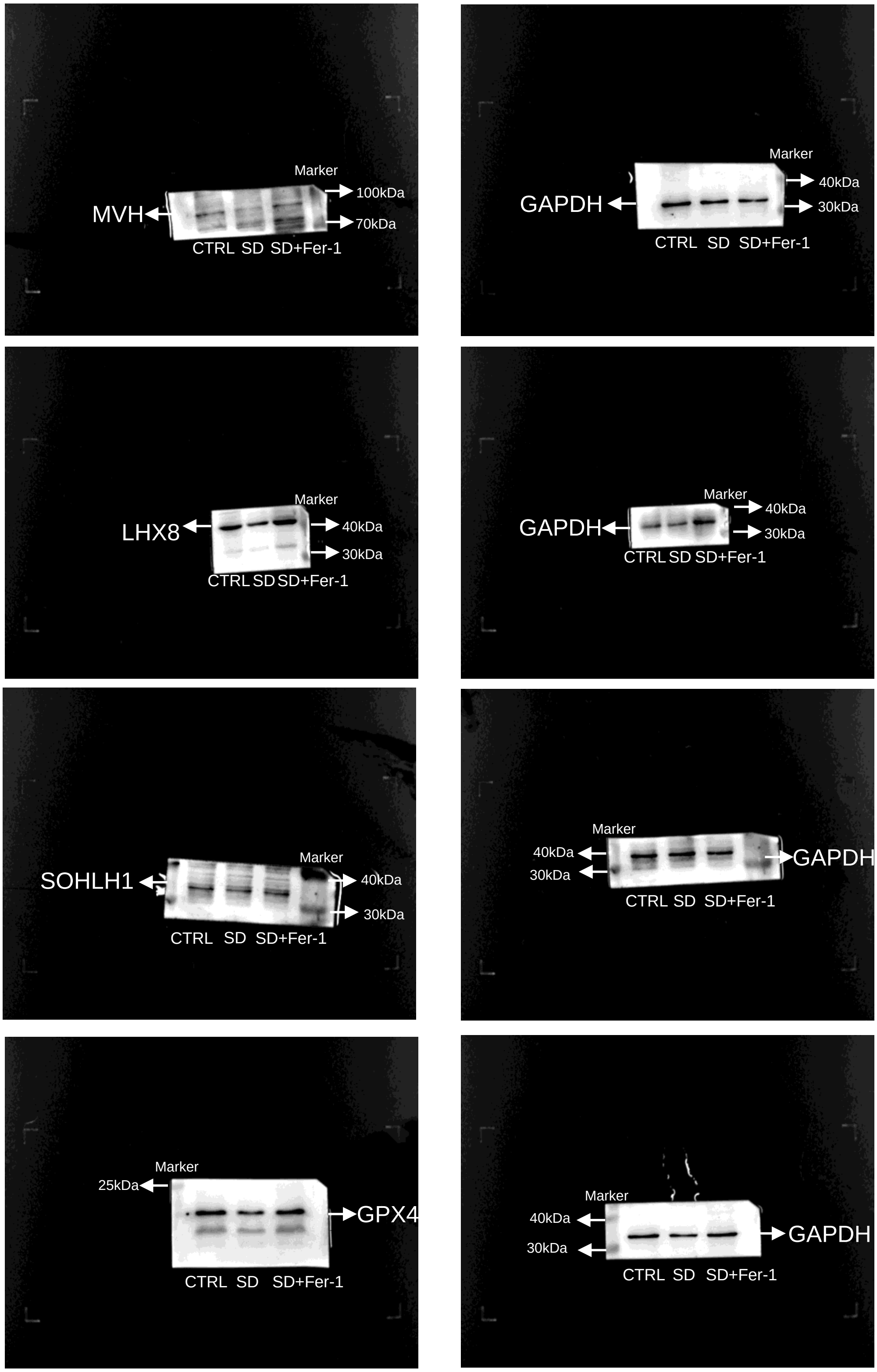

Marker protein used in WB experiment

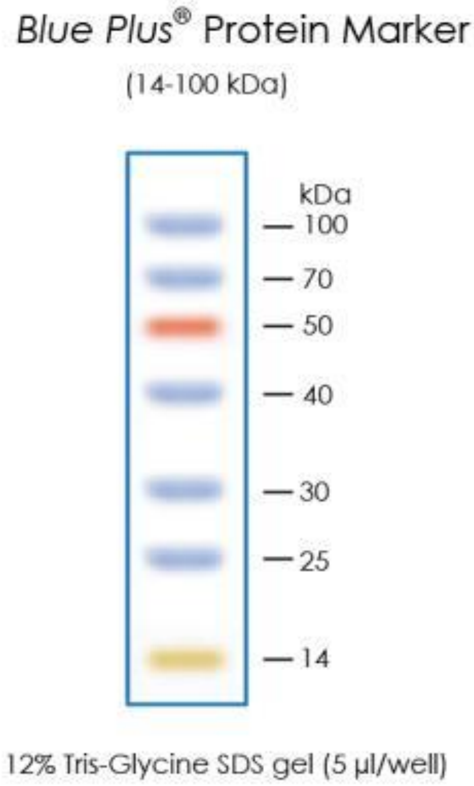

Supplement: Supplementary file 2 — Original western blots [file 41420_2025_2839_MOESM2_ESM.pdf]
